# Diptera in the Forensic Investigation of Human Deaths in Great Britain and the Dominant Role of *Calliphora vicina* (Calliphoridae) in Estimating a Minimum Post-Mortem Interval

**DOI:** 10.3390/insects17040422

**Published:** 2026-04-15

**Authors:** Martin J. R. Hall

**Affiliations:** Natural History Museum, London SW7 5BD, UK; m.hall@nhm.ac.uk

**Keywords:** blow fly, casework, diversity, suspicious death, *Calliphora vicina*, Calliphoridae, minimum post-mortem interval

## Abstract

Saprophagic flies can have a vital role in forensic investigations of suspicious death by providing an estimate of the minimum post-mortem interval (minPMI). An exemplar blow fly species, *Calliphora vicina* (Diptera: Calliphoridae), is frequently reported as being of greatest importance in this role, but without significant corroborating evidence. Therefore, the present study examined 122 anonymised, historic cases of suspicious death investigated in Great Britain (GB) to determine the role of Diptera and of *C. vicina* in particular. Thirty-seven fly species in eighteen families were recorded, with greater diversity outdoors (17 families) than indoors (11 families). Family diversity and the age of the oldest developmental stage increased as estimated minPMI increased. Indoor minPMIs tended to be lower than outdoor minPMIs, probably due to the higher likelihood of finding a body indoors. The most commonly collected family and species were Calliphoridae and *C. vicina*, respectively, the latter recorded in 69.7% (85/122) of all cases. The four main reasons for the importance of *C. vicina* are: (1) widespread distribution; (2) broad seasonality; (3) low thermal tolerance; and (4) ability to colonise bodies soon after death. It was used to determine minPMI in 85.9% of cases in which it was collected (73/85). Where a most likely PMI could be compared by regression analysis with a minPMI from *C. vicina* evidence, the relationship was robust (R^2^ > 0.98), accurate to within 2.5–2.7 days at a most likely PMI of 20 days. With greater research effort, *Calliphora vicina* and more neglected fly species can have an increasing role in future forensic investigations of suspicious death.

## Simple Summary

Carrion feeding flies can have a valuable role as forensic evidence when they naturally colonise human bodies in cases of suspicious death. The oldest developmental stages give the timing of body colonisation, the minimum post-mortem interval. The bluebottle blow fly, *Calliphora vicina*, is regarded as one of the most important insect indicators of minimum post-mortem interval, but there are few quantitative data that corroborate this. Therefore, the present study reports on the fly evidence recovered from 122 anonymised, historic cases of suspicious death in Great Britain. Diptera were collected from 93.4% of cases and were distributed between 18 fly families. The dominant family was the blow flies, found in 93.9% of cases with Diptera. *Calliphora vicina* was the dominant species both indoors and outdoors, used to estimate a minimum post-mortem interval in 59.8% of all cases studied. Where the minimum post-mortem interval from *C. vicina* could be compared with the most likely post-mortem interval, the relationship was robust, suggesting that *C. vicina* has great potential in future forensic investigations of suspicious death, both in Great Britain and worldwide. Greater research on *C. vicina* and more neglected species will increase the future value of insect evidence, especially in those cases where other evidence of post-mortem interval is unavailable.

## 1. Introduction

The larval feeding habits of blow flies (Diptera: Calliphoridae) are diverse, but all require a protein-rich substrate, with important examples being saprophages, which develop on carrion, and parasites of invertebrates or vertebrates [1]. The evolution of the parasitic lifestyle from a saprophagic habit, that is, moving to feeding on dead tissues on live animals (facultative parasitism) and ultimately to feeding only on live tissues (obligate parasitism), seems logical [2], but recent molecular studies indicate that larval saprophagy is not the ancestral calliphorid habit but is derived, being evolved more than once from ancestors that were larval parasites of invertebrates [3,4]. The carrion feeding behaviour of larval calliphorids can make them important forensic evidence at crime scenes involving human bodies [5].

A prime example of a carrion breeding calliphorid is the bluebottle blow fly *Calliphora vicina* Robineau-Desvoidy, 1830. This species is distributed worldwide, on all continents except Antarctica [6], and is the most common and widely distributed British species of *Calliphora* [7]. In keeping with its specific name, which means neighbour (derived from the Latin *vicinus*), *C. vicina* has a generally well-recognised, strongly synanthropic habit across its distribution [7,8]. However, it is very adaptable and can be found in many habitats [9], such that Greco et al. [10] reported its synanthropy as low (SI = 38.85), despite the fact that in their study of three ecological zones it was the dominant Calliphoridae species in urban areas (i.e., with >69% of specimens of six species; their Table 1 [10]), because it was also found in large numbers in their rural and wild areas. Nuorteva [11] considered that *C. vicina* was more synanthropic at the edges of its range in Europe. This synanthropy, by definition, brings it into close association with many aspects of human culture. Hence, it has been considered on the one hand to be a pest of food products [12], to be a nuisance to visitors in public buildings [13], to be a rare agent of myiasis of humans [14] and animals [15], and to be a possible mechanical vector of *Mycobacterium* as larvae and adults [16], although, in contrast, immature stages do not show a role as mechanical vectors of African swine fever virus [17]. On the other hand, the relationships of *C. vicina* with humans can be positive. For example, it can have a beneficial role in the pollination of crops [18,19], it makes an important contribution to nutrient recycling along with other calliphorids [20,21], and it is an aid in the forensic investigation of unexplained deaths, whether in countries where forensic entomology is regularly used (e.g., Germany [22]) or used for the first time (e.g., Iran, [23]). Indeed, the global value of *C. vicina* as a forensic indicator was highlighted in a study of 307 published case reports from around the world, which reported *C. vicina* as the insect species which had the greatest number of mentions and whose developmental data were used the greatest number of times (Table 1 in [24]).

*Calliphora vicina* is widely regarded as one of the most common and important species used as insect evidence in forensic investigations. In 1986, Smith wrote that, “*Calliphora vicina* is the commonest species found on human corpses, especially in urban situations…” ([25], p. 105). Writing more generally of bluebottle (*Calliphora*) species, Erzinçlioğlu ([26], p. 68) stated that the “…paragon of an entomological clue is the female bluebottle, together with her close relations”, while earlier he had written that *C. vicina* was “without doubt” the most important species in forensic cases in Britain [27]. However, few quantitative data from Great Britain (GB: England, Scotland and Wales) are available to verify this, despite the casework mentioned by both Smith [25] and Erzinçlioğlu [26] in their books. *Calliphora vicina* was certainly the species that was collected as evidence and provided a timing of body deposition that guided the investigation of the first case in GB that utilised forensic entomology techniques, the Ruxton case of 1935, in Moffat, Scotland [28].

There are surprisingly few reports of the diversity of forensically important Diptera in GB, either from trap studies [21,29,30,31,32] or from collections on animal parts or whole bodies [20,32,33], and few accounts of fly occurrence on human bodies [25,26,34], hence the latter was a focus of the present study. Hart et al. [35] discussed three case examples of the application of forensic entomology in the UK, two of which involved blow flies, and Hofer et al. [36] refer to the estimation of minimum post-mortem interval (minPMI) using *C. vicina* in one GB case. Forensic entomology estimates a minPMI because the age of an insect coloniser often underestimates the actual period since death, as it is not always possible to determine the duration of the period between death and initial insect colonisation, the pre-appearance interval (PAI). Under ideal conditions, the PAI can be short for early colonisers such as blow flies [36], but there can be significant delays in fly arrival due both to environmental factors, such as low springtime temperatures with limited fly availability [37,38], and to physical factors, such as a body being indoors [39,40], in a suitcase [41,42] or in the boot of a car [43]. Hence, the emphasis in forensic entomology casework on estimating a minPMI [44].

Using pigs as substitutes for human bodies, Turner and Wiltshire [45] validated forensic evidence from a human case in Southern England. Although pigs are a good analogue for human decomposition [46], they are not perfect [47] and, therefore, studies of insect diversity from humans, collected in forensic casework, have the potential to be of great value. Therefore, the present study examined the role of all Diptera collected, and of *C. vicina* in particular, in the forensic entomology investigations of 122 anonymised, historic cases of suspicious death in Great Britain, for which the author prepared forensic entomology reports as expert witness statements, objective and unbiased opinions on the insect evidence, submitted to either the prosecution or defence for presentation in court.

## 2. Materials and Methods

### 2.1. Case Selection and Geographical Area Covered

In the period 1990–2020, the author prepared expert witness statements on the insect evidence collected during 146 criminal investigations, 130 for the prosecution and 16 for the defence. Of the 146 cases, 23 did not concern dead bodies and one occurred outside GB, so these were removed, leaving a total of 122 anonymised, historic forensic case submissions examined here, including natural deaths, suicides and homicides. All cases were analysed with case-specific or other identifiable information removed. Insect species, distribution and seasonality were extracted from the witness statements and treated in a pooled manner.

Although the classification of a crime scene is complex [48], for simplicity here the scenes attended were considered based on their physical location as either indoor or outdoor [49]. Indoor scenes mainly involved bodies found in standard dwellings, houses or flats, but also garages or other outhouses, occasionally in a state of disrepair (e.g., doors or windows broken/missing), but always with a roof. Outdoor scenes were as the term implies, and even if a body was sometimes found next to the outside wall of a building, there was no roof covering it.

It is important to remember that the insect data collected were from forensic submissions rather than any systematic dipterological sampling of environments under controlled conditions. Therefore, the data collected may reflect the investigative context in addition to the ecological occurrence of Diptera species.

### 2.2. Collection of Evidence

Collection of insect evidence was undertaken in accordance with published protocols [50,51,52,53]. I visited the scenes to collect specimens in 22% (27/122) of the cases. In the remainder of cases insect evidence was collected by trained crime scene personnel, usually at the crime scenes but sometimes at forensic post-mortem examinations. The majority of specimens were hot water killed and preserved [54] at the time of collection, but a proportion were retained alive for rearing to adulthood, to assist in identification and, where possible, to assist in ageing [55]. Where I did not visit the scene, the evidence was couriered to me in exhibit bags which were signed for on both receipt and return, to retain a clear chain of evidence.

### 2.3. Identification of Evidence

Only morphological identification of evidence was made in the cases discussed here, by examination under a Leica M165C binocular microscope (Leica Microsystems, Wetzlar, Germany), or equivalent, at up to ×50 magnification. Identification was made by reference to voucher specimens of all developmental stages of flies in the extensive Diptera collections of the Natural History Museum and by using standard identification keys [25,56,57,58,59,60,61,62,63,64,65,66,67,68,69,70].

### 2.4. Determination of Developmental Temperatures

Environmental factors such as temperature are highlighted as challenges for the application of forensic entomology [71], and temperature has been described as the ‘weak point’ in forensic entomology [72]. Failure to accurately estimate the temperatures at which insects were developing on a body severely limits subsequent efforts to estimate the age of the insects, but even the placement of temperature data loggers at a scene to gather accurate data can be complex [36].

In most cases here a Tinytag temperature datalogger (Gemini Data Loggers, Chichester, UK) was placed at the scene of discovery of the body and left for up to ten days, depending on the need to release the scene. Climate data from the nearest weather station(s) were supplied by WeatherNet Ltd. (Bournemouth, UK). Measurements from the weather stations were taken in strict accordance with the World Meteorological Organisation (WMO) guidelines and the data underwent quality control at various stages to ensure that it was accurate and correct. For later cases, WeatherNet Ltd. also provided hourly temperatures, modelled (remote sensed) by the Meteorological Office (Exeter, UK) with a 2 km resolution, for the scene’s GB Post Code, based on all of the weather station data available to them.

### 2.5. Estimate of Insect Age and minPMI

The temperature data from the scene was compared with meteorological station data by linear regression analysis as discussed by Hofer et al. [73]. This enabled scene temperatures for the period before discovery of the body to be estimated and, thereby, the accumulated degree hours (ADH) for the developing insects to be calculated and the age of the insects estimated according to the thermal summation model [74]. Periods during evidence transport and in mortuary cold storage facilities were taken into account. Numerous published insect developmental data were used. That for the calliphorids *C. vicina*, *Lucilia sericata* (Meigen, 1826) and *Protophormia terraenovae* Robineau-Desvoidy, 1830 are summarised in Amendt et al. [50]. Additional data for *L. sericata* can be found in Wall et al. [75] and for *P. terraenovae* in Warren and Anderson [76]. Unpublished data for *C. vicina*, *C. vomitoria*, *L. sericata* and *Lucilia caesar* (Linnaeus, 1758) were also used (e.g., [77]).

In regression analysis of the minPMI estimated from analysis of *C. vicina* evidence against the most likely PMI, termed here the maxPMI after Hu et al. [24], only cases where the maxPMI from all other non-insect evidence was available were used (e.g., last confirmed sighting from CCTV evidence or witness statements, some cross-referenced with media sources [78]). Where minPMI was estimated as a range, then the midpoint value was used in the regression analysis (e.g., for an estimated minPMI range of 14–16 days, 15 days was the value used). Only minPMIs calculated up to one full developmental cycle, egg to emerging adult, were used as, clearly, if empty puparia were the oldest stages, then the period since adult emergence was usually unknown and the minPMI would very much underestimate the maxPMI. In addition, if diapause was a potential factor in a case at northern latitudes (see discussion in Section 3.4), the case was excluded.

Contextual information can introduce errors by expert witnesses into even standardised procedures such as fingerprint identification [79]. To minimise the possibility of cognitive bias in the estimation of minPMI, the default protocol was to estimate minPMI blind, with no knowledge of a likely PMI. Strict adherence to published guidelines [50] was followed, firstly to identify the insect evidence (Section 2.3), and secondly to age the insects based on estimated developmental temperatures (Section 2.4) and use of insect developmental data (Section 2.5). Since the primary objective of the forensic entomology investigation was to estimate a minPMI because PMI was unknown, in most cases blind analyses were the usual. However, in some cases, especially those of a high-profile nature with significant media attention, it could be impossible to avoid exposure to speculation of PMI from other evidence before the minPMI estimation was completed. Nevertheless, the same forensic entomology guidelines were followed in all cases to mitigate bias. Valuable approaches to reducing the impact of cognitive bias have now been published [80,81]. Examples of how adhering to guidelines reduced bias were two cases when non-insect evidence was presented before entomology analysis. Non-insect evidence gave initial PMIs of two days and of two weeks in the two cases, respectively, but the insect evidence later gave minPMIs of eight days and of three months, respectively. The insect-based estimates were subsequently accepted as providing the most accurate minPMIs.

### 2.6. Statistical Analyses

All statistical analyses and figure constructions were undertaken using Unistat Statistical Package Version 10.12 software.

## 3. Results and Discussion

### 3.1. Location and Seasonality of Body Discovery

Of the 122 cases of suspicious death in Great Britain, 49 (40.2%) were located at indoor scenes and 73 (59.8%) outdoors. Case scenes were widely distributed across GB, from the south of England to the lowlands of Scotland in the north, but particular foci, especially indoors, were within urban areas, where homicides are generally more common [82,83].

**Figure 1:**
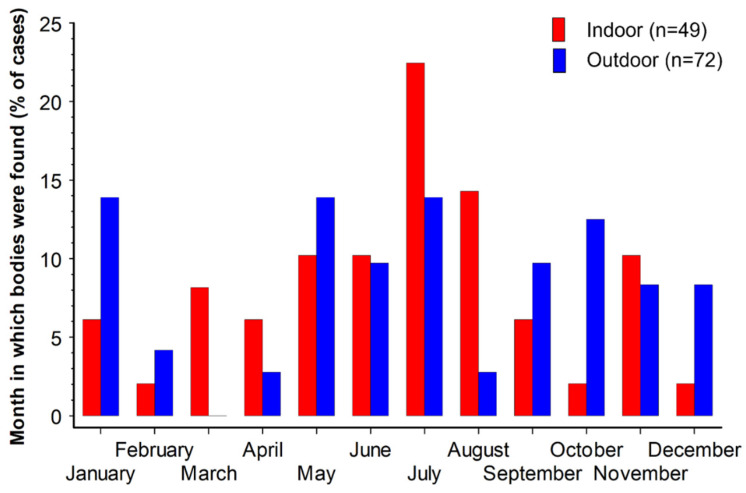
Month of discovery of bodies at indoor (red columns, n = 49) and outdoor scenes (blue columns, n = 72).

There was no record for the month of body discovery of one of the outdoor cases, but the monthly occurrence of discovery of the other 121 cases indicates that there was a slight emphasis on discovery of cases by investigators around the middle of the year (Figure 1). It is important to bear in mind that the seasonality represented here is not of deaths or of insect activity but is the seasonality of the discovery of bodies by investigators, from which insect specimens were recovered as evidence. Insect activity in GB is overall greatest in the summer season, but bodies are generally more likely to be found by investigators faster in indoor settings. This probably explains the summer season peak for discovery of bodies colonised by insects indoors, but a more drawn-out likelihood of discovery of bodies with insects outdoors into the autumn and winter (see also the discussion for calliphorids under Section 3.4 below). Analysing the data according to the UK season shows that the overall summer peak for case discovery was driven by indoor cases (Table 1). Outdoors, the proportion of case numbers was similar in summer, autumn and winter (26–30%), but with a much lower proportion in spring (16%). Indoors, the case numbers were clearly highest in summer, almost twice that of spring, followed by autumn and winter. The differences in the occurrence of cases across the four seasons as a proportion of all cases was significant between indoor and outdoor cases (Chi-square = 16.444, *p* = 0.0009), but not between indoor and all cases (Chi-square = 5.8643, *p* = 0.1184), nor between outdoor and all cases (Chi-square = 3.9910, *p* = 0.2624) (Table 1).

### 3.2. Occurrence of Fly Families in Cases with Diptera Collected

Diptera were collected at 93.9% (46/49) of indoor cases and 93.2% (68/73) of outdoor cases, proportions that were not significantly different (Chi-square = 0.0406, *p* = 0.8404). Of the three indoor cases with no Diptera (one in March, one in April, one in July), two were very recent deaths and the third involved a wrapped body in a box—none involved other insects. Of the five outdoor cases with no Diptera (one in January, one in April, two in May, one in October), three were very recent deaths; one involved a buried body and the fifth involved a body found in water. The latter outdoor case was the only one in this study from which only non-Dipteran insect evidence was recorded and this was considered adventitious. This evidence comprised larval cases of caddis flies (Trichoptera) and a single adult silver–green leaf weevil, *Phyllobius argentatus* Linnaeus, 1758 (Coleoptera, Curculionidae), but as this was the only case with only non-Dipteran evidence, it will not be considered further. All eight cases without Diptera were situations where insect arrival in general was challenging, not just fly arrival, either due to the limited period of availability (recent deaths) or to physical constraints at the location where the bodies were found.

**Figure 2:**
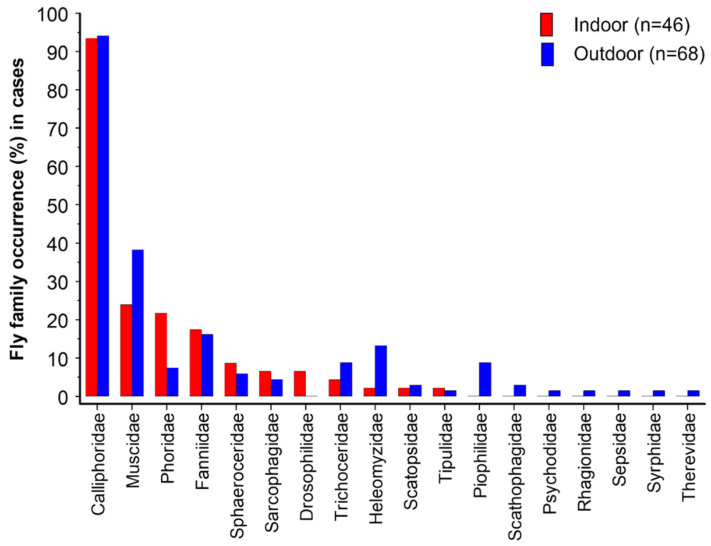
Occurrence of fly families at indoor (red columns, n = 46) and outdoor (blue columns, n = 68) scenes at which Diptera were collected as evidence.

Of the 18 fly families recorded, a greater diversity was recorded at outdoor scenes (17 families) than at indoor scenes (11 families, Figure 2). Lutz et al. [22] identified 13 fly families in their study of 279 bodies in Germany, while Hodecek et al. [84] identified 16 families in their study of 160 Swiss cases. Eleven of the families encountered by Lutz et al. ([22], their Table 3) and by Hodecek et al. ([84], their Table 1) were also found in this GB study (Figure 2), i.e., Calliphoridae, Muscidae, Phoridae, Fanniidae, Sphaeroceridae, Sarcophagidae, Drosophilidae, Trichoceridae (i.e., the first eight families from the left of Figure 2), Piophilidae, Sepsidae and Syrphidae. These eleven families therefore represent the most common flies found on human bodies in Europe. Scatopsidae was reported by Lutz et al. [22] and by this study, while Heleomyzidae was reported by Hodecek et al. [84] and this study. The family Stratiomyidae was unique to the German study; the four families Lauxaniidae, Dryomyzidae, Anthomyiidae and Chironomiidae were unique to the Swiss study; and the five families Tipulidae, Scathophagidae, Psychodidae, Rhagonidae and Therevidae were unique to the GB study: all of those ten families unique to a single study were found in only a very small number of cases.

As in Lutz et al. [22], for both indoors and outdoors the dominant family was unequivocally Calliphoridae, which were found in more than 93% of cases with Diptera, more than twice the proportion of the next most common family, Muscidae (Figure 2). Considering all cases with Diptera, the proportion with Calliphoridae (93.9%, 107/114) was significantly higher than that with Muscidae (32.5%, 37/114) (Chi-square = 89.7, *p* < 0.0001). These proportions were similar to those recorded in Switzerland, 91.8% (145/158) for Calliphoridae and 26.6% (42/158) for Muscidae of 158 cases where necrophagous flies were present [84]. Specimens of the families Calliphoridae and Muscidae also dominated the insects collected from 356 cases in France and its overseas territories [85]. The Phoridae were shown to be an important family in all studies, the third most numerous indoors here, especially as their small size can enable access to scenes that larger flies are excluded from. They can be difficult to identify, but the work of Disney ([86] and subsequently) has stimulated studies to enable their use increasingly in forensic cases [87,88,89].

Many of the families that were collected in a lesser proportion of cases than Calliphoridae have factors associated with them that make their use as forensic evidence more difficult, especially the inability to identify them to species level as larvae and their lack of developmental data. Nevertheless, those factors do not mean that they will not have future value in forensic investigations. Muscidae, as an example, are a family in which much progress has been made recently, both in identification (e.g., [70]) and in studies of their development (e.g., [90,91]). Matuszewski and Mądra-Bielewicz [55] found published developmental data for the Piophilidae, *Stearibia nigriceps*, to be accurate in estimating minPMI, but data is only available for pupariation and eclosion, illustrating the need for more extensive developmental studies across many species.

**Figure 3:**
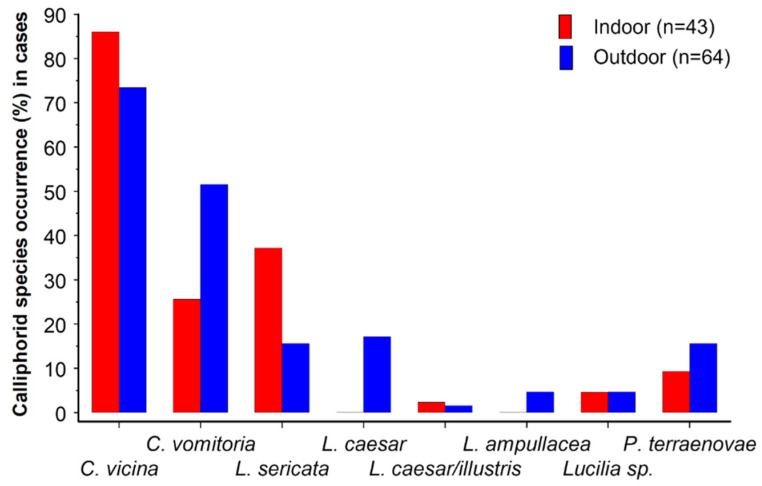
Occurrence of species of Calliphoridae at indoor (red columns, n = 43) and outdoor (blue columns, n = 64) scenes at which Calliphoridae were collected as evidence (*C* = *Calliphora*; *L* = *Lucilia*; *P* = *Protophormia*).

In total, six Calliphoridae species (Figure 3) and 31 non-Calliphoridae species were identified (Table 2). Sometimes immature stages were reared to the adult stage to aid in identifying the species, but only the immature stage collected was listed. Clearly species collected only as adults might not have laid eggs on the body at the scene, and for some species collection of larvae does not imply they had developed on the body, as they might have been adventitiously included in a soil sample collected from around a scene (e.g., Tipulidae) together with dispersing necrophagous larvae. Sometimes more than one species in the genus were collected from the same scene. However, cases with a known species are only included under that species name, not under “*species* sp.”. For example, for the genus *Megaselia* in the family Phoridae, there were six indoor cases where the species of *Megaselia* was listed as “sp.” as it could not be identified, but there were also two indoor cases with *M. rufipes* identified, therefore eight indoor cases in total in which the genus *Megaselia* was collected.

Of the Muscidae genera, despite their appearance at many outdoor cases, no *Hydrotaea* species were collected here from indoor cases. Lutz et al. [22] stated that *Hydrotaea* showed a clear preference for outdoor crime scenes. The most encountered species of *Hydrotaea* here was *H. dentipes*, the second most identified species by Lutz et al. [22] and by Hodecek et al. [84]. *Hydrotaea similis* was only first reported to be able to develop on a dead human body in 2014 [68] and it is likely that the two records here represent the first report from GB. Doubtless, further case reports will identify other species of Diptera previously unreported as colonisers of human bodies.

### 3.3. Occurrence of Calliphoridae Species in Cases with Calliphoridae Collected

Calliphoridae were collected at 93.5% (43/46) of cases when Diptera were collected indoors. The three cases without Calliphoridae but with other families of Diptera involved a mummified body (with *Fannia* [Diptera: Fanniidae] and *Dermestes* [Coleoptera: Dermestidae]), a body buried in concrete (with *Conicera* [Diptera: Phoridae]) and a case of myiasis (with *Musca domestica* [Diptera: Muscidae]). Calliphoridae were collected at 94.1% (64/68) of cases with Diptera outdoors. The four cases without Calliphoridae but with other Diptera all involved buried bodies (one with Heleomyzidae, one with Sphaeroceridae, one with Phoridae and Sphaeroceridae and one with Heleomyzidae, Therevidae and Tipulidae). All seven cases without Calliphoridae involved situations where Calliphoridae would not normally be encountered, i.e., on a mummified body indoors, inside concrete without access to larger Diptera, in a case of myiasis, to which *M. domestica* were most likely initially attracted by odours of faecal material, before the victim’s death due to several compounding factors, and buried (×4 cases). Although blow flies have been shown to survive burial in soil and to emerge as adults from as deep as 50 cm [93], a shallow burial in just 20 cm of soil can deter colonisation of pig liver by *C. vicina*, *C. vomitoria* and *L. sericata* [94] and a 25 cm burial prevented calliphorid colonisation of pigs [95].

*Calliphora vicina* was clearly the dominant species at both indoor and outdoor scenes in GB (Figure 3), being found at 86.0% (37/43) and 73.4% (47/64) of indoor and outdoor cases, respectively, at which species of Calliphoridae were recovered. *Calliphora vomitoria* [96] was the second most common species at outdoor scenes, being recorded outdoors in three times as many cases as indoors, reflecting the observation of Lutz et al. [22] that it tended to appear more frequently on bodies located outdoors. *Lucilia sericata* was the second most common species here at indoor scenes. *Lucilia caesar* was not found indoors and was uncommon outdoors, although specimens grouped as *L. caesar*/*illustris* were recovered from one indoor scene. Lutz et al. [22] reported that *L. caesar* showed a clear preference for outdoor scenes. *Protophormia terraenovae* was similarly uncommon here at both indoor and outdoor scenes. Species descriptions and data on distributions, seasonality and biology of all five common calliphorids collected are provided in [97,98].

In a study in Germany, Lutz et al. [22] found 39.8% of 279 human bodies were colonised by *C. vicina*. Similarly, in a study in Central Europe, Bernhardt et al. [99] found *C. vicina* on 43.1% of 51 human bodies, an equal number to those colonised by *Protophormia terraenovae* and very similar to *Lucilia ampullacea* Villeneuve, 1922 (45.1%). In both studies, the dominant calliphorid species was *L. sericata*, 53.8% in Germany and 86.3% in Central Europe. In a trapping study in Sicily, the most collected blow fly species was *L. sericata* (68.5%) with *C. vicina* second (21.0%) [100], but the proportions differed between altitudes along a gradient. Conversely, in a study of casework in Switzerland, Hodecek and Jakubec [101] found that the most frequently occurring calliphorid species was *C. vicina*, found in 69.0% of cases colonised by blow flies (100/145), compared to *L. sericata* in 30.3% of cases (44/145). The Swiss data most closely matched those of GB.

*Calliphora vicina* was identified among the insect samples in 75.5% (37/49) of all indoor cases and 65.8% (48/73) of all outdoor cases, i.e., overall in 69.7% (85/122) of all cases. In the 12 indoor cases where *C. vicina* was not collected, the likely reasons were either: (1) recent death with no insects (×2 cases); (2) blow fly access restricted due to house construction, body packaging or burial in concrete (×4 cases); or (3) no obvious reason as other calliphorids/muscids were present (×6 cases). In the 25 outdoor cases where *C. vicina* was not collected, the likely reasons were either: (1) recent death with no insects (×3 cases); (2) blow fly access restricted due to submersion in water or burial in soil (×8 cases); or (3) no obvious reason as other calliphorids/muscids were present (×14 cases, possibly due to sub-optimal environments for *C. vicina*, e.g., body found in woodland).

### 3.4. Distribution and Seasonality of Calliphorid Species

All calliphorid species were collected from locations within the generally well-known distributions for Calliphoridae in the UK [97,98]. No new distributional data were produced; therefore, to maximise anonymity the locations are not documented here. *Calliphora vicina* was the most widely distributed species in both indoor and outdoor scenes. *Calliphora vomitoria* was similarly well distributed, but mostly at outdoor scenes, as already mentioned. *Lucilia sericata*, *L. caesar* and *P. terraenovae* were less well distributed, as published data recognises [97,98], with *L. caesar* found only at outdoor scenes.

**Figure 4:**
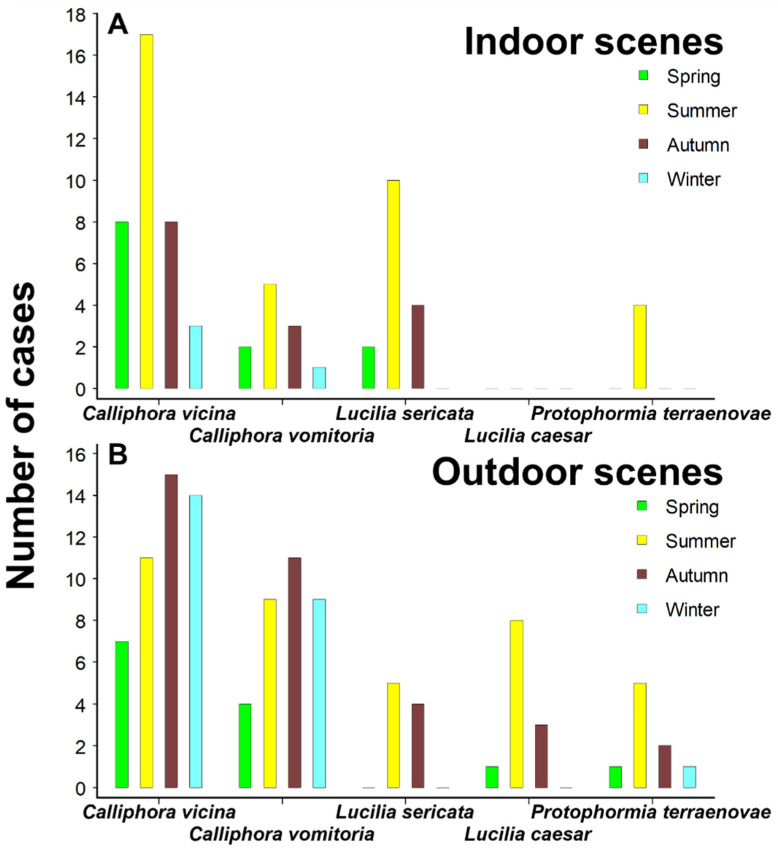
Seasonality of collection of the five most numerous Calliphoridae species (*C* = *Calliphora*; *L* = *Lucilia*; *P* = *Protophormia*), based on the numbers of indoor (**A**) and outdoor (**B**) scenes they were collected at from bodies first discovered in each of the four GB seasons, i.e., spring (March–May, green columns), summer (June–August, yellow columns), autumn (September–November, brown columns) and winter (December to February, blue columns).

All four species of Calliphoridae that were found at indoor scenes were found most frequently at cases discovered in the summer season (Figure 4A). *Calliphora vicina* and *C. vomitoria* were found at indoor cases in all four seasons, but the seasonality of investigator discovery of bodies with *L. sericata* and *P. terraenovae* was more limited, being dominated by summer cases.

**Figure 5:**
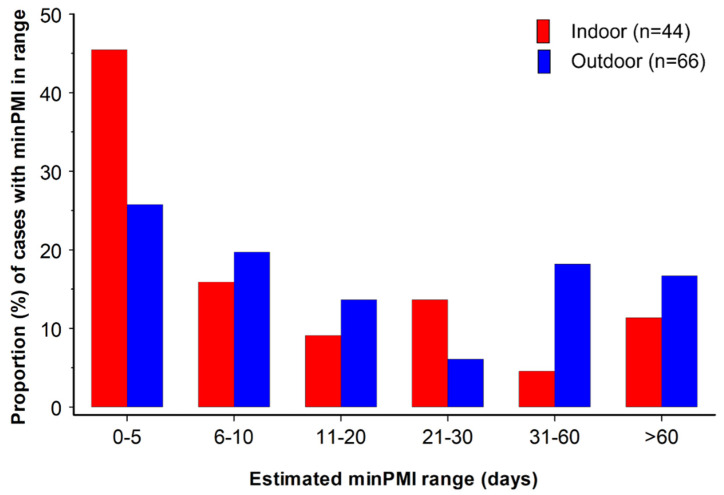
Proportion of indoor (red columns, n = 44) and outdoor (blue columns, n = 66) cases at which a minimum post-mortem interval (minPMI) within the ranges given could be estimated. Note that the minPMI ranges are not equal.

Summer was also the peak season for investigator discovery of bodies colonised by *L. sericata*, *L. caesar* and *P. terraenovae* at outdoor scenes (Figure 4B). However, for both *C. vicina* and *C. vomitoria*, the peak season for their discovery on bodies outdoors was the autumn, and even the winter season had as many or more cases for those species as the summer season. For all species, the season of discovery at outdoor scenes in which the fewest bodies were found colonised was spring. Similarly to Bourel et al. [37], Martín-Vega et al. [102] observed that insect successional patterns might be much more unpredictable in spring than at other times of year, and that changeable spring weather might make a potential estimation of the season of death difficult, when based on the composition of insect species recorded on a body in spring. General calliphorid flight activity in GB is overall greatest in the summer season [97,98], but bodies tend to be found faster in indoor settings (see below and Figure 5). This probably explains the summer season peak for discovery of bodies colonised by calliphorids indoors, but with a more drawn-out likelihood of discovery of bodies with calliphorids outdoors into the autumn and winter.

Clearly with long-term changes in climate and land cover, plus species introductions, forensic entomologists need to be alert to the possibility of changes in the abundance, seasonal flight activity and distribution of important forensic indicators like blow flies [103,104,105,106]. It is apparent that *C. vicina* has a different seasonality at different latitudes and altitudes, for example, in a trapping study in central Spain, at two lower level zones it was found to be a dominant species in autumn, winter and spring but uncommon in summer, whereas, at a third, higher level zone it was most abundant in summer, and was described as thermophobic (Tables 2–5 in [107]), in contrast to the thermophilic species, *Lucilia sericata* and *Chrysomya albiceps* (Wiedemann, 1819). In northern Spain *C. vicina* was most abundant in traps in Spring, but summer was the season of the second greatest abundance [108]. However, at the northern latitudes of Norway, *C. vicina* was clearly most abundant in traps at stockfish production areas in the summer season [109]. Accepting the limitations of a single year of study, in Germany, Lutz et al. [110] trapped adults of *C. vicina* throughout their study period, April to October, although fewer flies were found in the warmer summer months than in spring or autumn. In contrast, the summer months were dominated by *L. sericata*, which was almost absent in spring and autumn. Hutchinson [30] also conducted a single year study, January to October, of blow fly availability on traps in southern GB. All four species, *C. vicina*, *C. vomitoria*, *L. caesar* and *L. sericata* had single summer peaks in abundance, but the peaks for the *Lucilia* species were much narrower, while those of the *Calliphora* species were much wider, spread across the year. Interestingly, the increase in abundance for both *Calliphora* species occurred a month earlier in urban areas than in rural areas. This could be another reason for the earlier collection of *Calliphora* species in indoor cases than in outdoor cases (Figure 4), as indoor cases are more likely to be located in urban than rural settings.

The possibility that there had been changes in species composition on bodies during the 30-year period over which casework data were collected here was studied by dividing the cases into two 15-year periods, 1990–2004 and 2005–2019. After removal of one indoor case with no species identification and two outdoor cases either without a date or with a date outside the year ranges, 41 cases in the earlier period and 63 in the later period were considered. In each period the proportion of indoor/outdoor cases was very similar, i.e., 39.0%:61.0% respectively in 1990–2004 and 41.3%:58.7% respectively in 2005–2019. Although there appeared to be a slight rise in the proportion of cases colonised by the four less numerous species in the later period, the distribution of colonisation numbers was not significantly different between periods (Table 3; Chi-square = 1.6619, *p* = 0.7976). The proportion of cases at which *C. vicina* was collected was not significantly different for the earlier (80.5%) and later (79.4%) periods (Chi-square = 0.0316, *p* = 0.8590; Table 3). Therefore, over the 30-year period studied here, there did not appear to be any significant effect of long-term environmental changes on the calliphorid species assemblages colonising bodies.

While blow flies such as *C. vicina* are available almost year round in southern UK and do not usually enter the hibernation-like stage called diapause [111], diapause is a feature of populations of *C. vicina* in northern GB and requires increasing daylight to break it. Saunders [112] demonstrated that diapause is more intense with more northerly populations of *C. vicina* when he compared populations from Italy, Scotland and Finland. It is likely that populations of *Calliphora* in Scotland have a longer period of overwintering and appear later in the year than they do in southern GB.

### 3.5. Estimation of Minimum Post-Mortem Interval

Where minPMI could be measured, it tended to be lower at indoor scenes than at outdoor scenes (Figure 5). Thus, minPMI was in the range of 1–5 days or >30 days in 45.5% (20/44) and 15.9% (7/44), respectively, for indoor cases, but in the same minPMI ranges in 25.8% (17/66) and 34.8% (23/66), respectively, of outdoor cases. The difference in distribution of cases between the six categories of minPMI (Figure 5) at indoor and outdoor scenes was significant (Chi-square = 17.0237, *p* < 0.005). The lower indoor minPMIs probably reflect the greater likelihood of finding a body sooner when it is located indoors than when it is found outdoors, for example, due to neighbours alerting legal authorities of a malodour.

**Figure 6:**
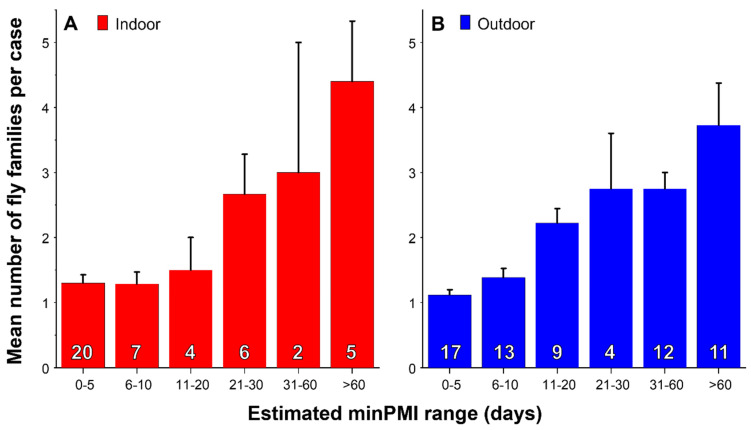
Mean number of fly families collected at indoor ((**A**), red columns, n = 44) and outdoor ((**B**), blue columns, n = 66) cases where the minimum post-mortem interval (minPMI) could be estimated. The number of cases in each minPMI range from which the means were estimated is shown inside the columns in white letters. Bars represent 95% confidence limits. Note that the minPMI ranges are not equal.

**Figure 7:**
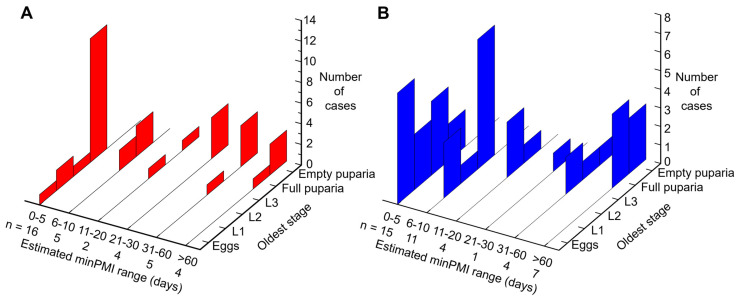
Number (n) of indoor cases (**A**, red columns, 36 cases) and outdoor cases (**B**, blue columns, 42 cases) with indicated minPMI (days) measured only by the collected *Calliphora vicina* evidence at which the oldest developmental stage was either eggs, L1 (first instar larvae), L2, L3, full puparia or empty puparia. Note that the minPMI ranges are not equal.

For both indoor and outdoor cases the biodiversity of Diptera, measured by the number of colonising fly families collected on the body, increased as estimated minPMI increased (Figure 6), reflecting the succession of insects on a body, with the greater availability of ageing bodies to colonising Diptera that are attracted to increasingly advanced stages of decay [113]. As would be expected, the oldest developmental stages of *C. vicina* collected at any scene, both indoor and outdoor, increased as the estimated minPMI increased (Figure 7), with eggs being the oldest stage only at minPMIs of up to 5 days and puparia, full or empty, being the only stages found at minPMIs greater than 60 days.

### 3.6. Use of Calliphora vicina to Estimate Minimum Post-Mortem Interval

Where *C. vicina* was collected, it was used to estimate minimum post-mortem interval (minPMI) in 89.2% (33/37) and 83.3% (40/48) of indoor and outdoor cases, respectively, i.e., in 85.9% (73/85) of all cases where *C. vicina* was collected. This equates to its use as the main indicator of minPMI in 67.3% (33/49) and 54.8% (40/73) of all indoor and outdoor cases respectively, i.e., in 59.8% (73/122) of all cases.

In cases where minPMI was estimated by reference to the development of *C. vicina*, the minPMIs were compared to the maxPMI (i.e., the most likely PMI) from all other non-insect forensic evidence. This was possible in only 27.3% (9/33) of indoor cases and 45.0% (18/40) of outdoor cases. The proportion of outdoor cases with both a minPMI and a maxPMI was similar to the 42.0% of cases with a minPMI and maxPMI in the casework analysis of Hu et al. [24].

In 40.7% (11/27) of all cases where a maxPMI was available, analyses might not have been blind as an indication of maxPMI was included in the expert witness statements. The time during the analysis in which this information was made available was not recorded, but measures to mitigate cognitive bias were followed in all cases (Section 2.5).

**Figure 8:**
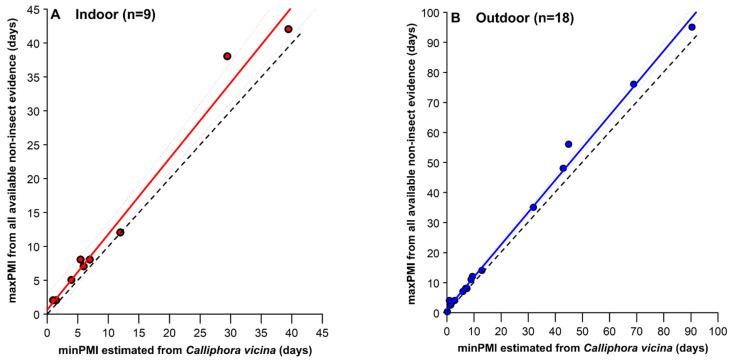
Comparison of minPMI from *Calliphora vicina* evidence (x-axis) versus maxPMI from all available non-insect evidence (y-axis). (**A**): Indoor cases (n = 9), red regression line, R^2^ = 0.9821, y = 1.1164x + 0.6291. (**B**): Outdoor cases (n = 18), blue regression line, R^2^ = 0.9953, y = 1.0753x + 1.1570. The dashed coloured lines represent 95% confidence limits for the mean of y. On both graphs the dashed black lines represent the situation where maxPMI = minPMI (i.e., y = x).

The linear regression analyses gave high values of the coefficient of determination (R^2^) for both indoor (Figure 8A; R^2^ = 0.9821) and outdoor (Figure 8B; R^2^ = 0.9953) situations, with maxPMI values always being at least equal to but generally greater than minPMI, due to the delay in colonisation of bodies and uncertainty over actual time of death, such that maxPMI can be a time before actual death. The indoor and outdoor data were kept separate here, but analysis of covariance (heterogeneity of regression) tests showed that the indoor and outdoor regressions were not significantly different, neither by slope (F = 0.5187, *p* = 0.4787) nor intercept (F = 0.0001, *p* = 0.9916).

Although the numerical difference between maxPMI and minPMI increased as maxPMI increased, also reported by Hu et al. [24], this difference as a proportion of the maxPMI decreased, both in indoor and outdoor cases. Hence, when maxPMI = 10 days, the minPMI for indoor and outdoor cases, respectively, were 1.6 and 1.8 fewer days, differences equivalent to 16% and 18% of the maxPMI (from the regression formula for Figure 8A). However, when maxPMI = 40 days, the minPMI for indoor and outdoor cases, respectively, were 4.7 and 3.9 fewer days, differences equivalent to 12% and 10% of the maxPMI (from the regression formula for Figure 8B). Although the present study and that of Hu et al. [24] are not directly comparable in terms of maxPMI and minPMI, i.e., minPMI was calculated here from *C. vicina* in GB alone, whereas minPMI in Hu et al. [24] was calculated from multiple insect species worldwide, the mean values of maxPMI-minPMI were similar between the two studies. Hence, at maxPMIs of 10, 20 and 40 days, the mean values of maxPMI-minPMI were 1.8, 2.5 and 3.9 days for outdoor cases here and approximately 1.0, 2.5 and 6.0 days for the Hu et al. data ([24]; their Figure 9a). In terms of the Estimation Accuracy Index (EIA = [maxPMI-minPMI]/maxPMI) of Hu et al. [24], for maxPMIs of 10, 20 and 40 days, the maxPMI-minPMI values equate to EIAs of 0.18, 0.13 and 0.10 for outdoor cases here and approximately 0.10, 0.13 and 0.15 for the Hu et al. [24] data.

The strong relationship between minPMI and maxPMI across a wide variety of casework in different seasons and locations in GB gives strong support to the use of *C. vicina* developmental data for estimating minPMI, and thereby maxPMI, in forensic investigations in GB and elsewhere, especially given that there are still cases, as here, where no other means to estimate PMI exist. The data also suggest that, with a greater number of casework data to validate the approach, the analysis of maxPMI against estimated minPMI can be a method for addressing the issue of pre-appearance interval [44,114], although caution should always be exercised due to the complexity of each individual crime scene.

Where *C. vicina* was collected but not used to indicate minPMI, other calliphorid or muscid species were collected that were older and had, therefore, found the body sooner than *C. vicina* and took precedence in estimating minPMI: indoors (×4 cases) these were *C. vomitoria* (×2 cases), *L. sericata* (×1 case) and *P. terraenovae* (×1 case); outdoors (×8 cases) these were *C. vomitoria* (×4 cases), *L. sericata* (×1 case), *P. terraenovae* (×1 case) and *Muscina prolapsa* (×2 cases). For the four indoor cases, no maxPMI was available, but it was available for four of the outdoor cases. In three of those cases only one or two specimens of *C. vicina* were collected, insufficient for a robust analysis of minPMI, but in one case a minPMI of 15–17 days was estimated from *C. vicina* compared to the maxPMI of 21 days. At this wooded, rural scene, a closer minPMI came from the analysis of *C. vomitoria* evidence, which gave a minPMI of 17–20 days. Hwang and Turner [31] showed that along a survey transect from central, urban London to the rural surroundings, *C. vicina* characterised the urban environment and *C. vomitoria* characterised the rural woodland habitat, so earlier arrival of *C. vomitoria* than *C. vicina* in a woodland setting would fit with their ecology. *Calliphora vomitoria* is also a more slowly developing species than *C. vicina* [115].

## 4. Conclusions

With the use of *C. vicina* as the main provider of an estimate of minPMI in almost 60% of all cases reported here, including cases where no insects were collected, and in 86% of cases where *C. vicina* was collected, it is clear that *C. vicina* was the most used insect forensic indicator of minPMI in GB. In those cases where a likely PMI could be determined from non-insect evidence, the minPMI provided by *C. vicina* was, on average, accurate to within <3 days at a most likely PMI (maxPMI) of 20 days, at both indoor and outdoor scenes. The relationship between minPMI and maxPMI was very robust (Figure 8), providing compelling evidence of the value to forensic investigations of the use of minPMI to estimate PMI in those cases where evidence from *C. vicina* is the only evidence available, and of its corroborative support where other evidence is available.

It is certain that *C. vicina* and other calliphorids will continue to have a vital role to play in forensic investigations of suspicious deaths as their evidence can provide a time frame that has been shown to support other evidence, focus limited human resources especially at an early stage in investigations (e.g., in directing periods for CCTV observations), and can lead to suspect plea changes from not guilty to guilty [116]. More rarely, it can provide information in cases of neglect, when a myiasis infestation is initiated before death. The four main reasons for its forensic importance in GB are: (1) its widespread distribution, especially in urban settings [97,117]; (2) its broad seasonality (Figure 4; [97,101,118]); (3) its ability to develop at low temperatures [119]; and (4) its ability to colonise bodies soon after (within 1–3 h of exposure in the environment, [36]) or even before death [14,120]. However, those same reasons can be extended to its worldwide distribution, for example, in such diverse locations as Argentina, where it is the most common species in winter months [121] and India, where it can also be a winter species on pig bodies [122].

Towards the end of the 19th century, Lowne [123,124], working at the Middlesex Hospital Medical School in London, provided the first monograph on *C. vicina*. His work focussed on blow fly anatomy and physiology and is still relevant today [125], but he also commented on the carrion-seeking behaviour of gravid females, which led to this species first forensic role in GB just ten years after Lowne’s death [28]. Further such studies of the biology and behaviour of *C. vicina* will enhance its use in future forensic investigations, not only in GB but worldwide, as it has been the number one insect species mentioned in a survey of global forensic entomology cases [24]. In addition, similar research is needed on other species of Diptera encountered in forensic investigations, especially other Calliphoridae and Muscidae, for which developmental data are currently limited or unavailable and yet are frequently encountered at investigations of human death. Studies are also needed on those species which arrive later in succession on the body and have the potential, therefore, to provide evidence of period since death beyond the single egg to emerging adult developmental period of *C. vicina* and other early colonisers.

## Figures and Tables

**Table 1:** Seasonality of discovery of bodies in GB casework, indoors, outdoors and totalled (n = number of cases; % = n as a percentage of total).

Season	Indoor		Outdoor		All Locations	
	n	%	n	%	n	%
Winter (Dec–Feb)	5	10.2	19	26.4	24	19.8
Spring (Mar–May)	12	24.5	11	15.3	23	19.0
Summer (Jun–Aug)	23	46.9	20	27.8	43	35.5
Autumn (Sep–Nov)	9	18.4	22	30.6	31	25.6
Totals	49	100	72	100	121	100

**Table 2:** Alphabetical listing of non-Calliphoridae Diptera collected as part of forensic evidence. Numbers indicate the number of cases at indoor or outdoor scenes from which the species listed were collected. Where species were only identified to genus, the taxonomic authority given is that for the genus. All stages of each species at the time of collection are listed; sometimes more than one stage was collected at one scene (L = larvae, P = puparia, A = adult). All taxa marked with an asterisk (*) and the five Calliphoridae species discussed in the text are illustrated with a brief description of biology and distribution in GB in [92].

Family	Species	Authority	Numbers of Cases and Evidence Stages	
			In Door	Out Door
* Drosophilidae	*Drosophila* sp.	Fallen, 1823	1 (P)	0
	*Drosophila busckii*	Coquillett, 1901	1 (A)	0
* Heleomyzidae	*Tephrochlamys* sp.	Loew, 1862	0	1 (L)
	*Tephrochlamys rufiventris*	(Meigen, 1830)	0	1 (L)
* Fanniidae	* Fannia armata	(Meigen, 1826)	0	1 (A)
	* Fannia canicularis	(Linnaeus, 1761)	4 (LPA)	1 (L)
	*Fannia manicata*	(Meigen, 1826)	1 (A)	8 (LP)
	*Fannia scalaris*	(Fabricius, 1794)	4 (LPA)	8 (LPA)
* Muscidae	* Graphomya maculata	(Scopoli, 1763)	0	1 (A)
	* Hydrotaea sp.	Robineau-Desvoidy, 1830	0	12 (L)
	* Hydrotaea cyrtoneurina	(Zetterstedt, 1845)	0	1 (A)
	*Hydrotaea dentipes*	(Fabricius, 1805)	0	8 (LP)
	* Hydrotaea ignava	(Harris, 1780)	0	1 (A)
	*Hydrotaea similis*	Meade, 1887	0	2 (LA)
	* Musca domestica	Linnaeus, 1758	2 (LP)	0
	* Muscina sp.	Robineau-Desvoidy, 1830	4 (LP)	4 (LP)
	* Muscina levida	(Harris, 1780)	0	1 (P)
	* Muscina prolapsa	(Harris, 1780)	3 (LA)	4 (LPA)
	* Muscina stabulans	(Fallén, 1817)	3 (PA)	2 (L)
* Phoridae	*Conicera tibialis*	Schmitz, 1925	1 (LP)	0
	* Megaselia sp.	Rondani, 1856	6 (LPA)	1 (P)
	*Megaselia rufipes*	(Meigen, 1804)	2 (LP)	1 (P)
	*Diplonevra florescens*	(Turton, 1801)	0	1 (L)
	*Triphleba hyalinata*	(Meigen, 1830)	0	1 (L)
* Piophilidae	*Liopiophila varipes*	(Meigen, 1830)	0	1 (A)
	*Stearibia nigriceps*	(Meigen, 1826)	0	4 (LPA)
* Sarcophagidae	* Sarcophaga sp.	Meigen, 1826	1 (L)	0
	*Sarcophaga argyrostoma*	(Robineau-Desvoidy, 1830)	2 (L)	1 (L)
* Scathophagidae	* Scathophaga sp.	Meigen, 1803	0	1 (L)
	* Scathophaga stercoraria	(Linnaeus, 1758)	0	1 (A)
* Scatopsidae	*Scatopse* sp.	Geoffroy, 1762	0	2 (L)
* Sepsidae	*Meroplius minutus*	(Wiedemann, 1830)	0	1 (A)
	* Nemopoda nitidula	(Fallén, 1820)	0	1 (A)
* Sphaeroceridae	*Leptocera* sp.	Olivier, 1813	0	1 (L)
	*Phthitia empirica*	(Hutton, 1901)	1 (P)	1 (LPA)
* Stratiomyidae	* Sargus bipunctatus	(Scopoli, 1763)	0	1 (L)
* Syrphidae	* Eristalis intricaria	(Linnaeus, 1758)	0	1 (L)
* Therevidae	* Thereva plebeja	(Linnaeus, 1758)	0	1 (L)
* Tipulidae	* Tipula paludosa	Meigen, 1830	0	1 (L)
	* Tipula vernalis	Meigen, 1804	0	1 (L)
* Trichoceridae	* Trichocera sp.	Meigen, 1803	1 (L)	3 (L)

**Table 3:** Comparison between two successive 15-year periods to demonstrate consistency of calliphorid species assemblages over the entire study period. Proportion of all GB cases, indoor and outdoor, involving Calliphoridae in which the five most common species were collected as evidence (n = number of cases in which species were collected; % = proportion of all cases in each period in which species were collected). See Section 3.4 for discussion.

Period	All Cases		*C. vicina*		*C. vomitoria*		*L. sericata*		*L. caesar*		*P. terraenovae*	
	n	%	n	%	n	%	n	%	n	%	n	%
1990–2004	41	100	33	80.5	15	36.6	8	19.5	4	9.8	4	9.8
2005–2019	63	100	50	79.4	29	46.0	17	27.0	7	11.1	10	15.9

## Data Availability

The original contributions presented in this study are included in the article. Further inquiries can be directed to the corresponding author.
